# A clinical-molecular update on azanucleoside-based therapy for the treatment of hematologic cancers

**DOI:** 10.1186/s13148-016-0237-y

**Published:** 2016-06-21

**Authors:** Jeannine Diesch, Anabel Zwick, Anne-Kathrin Garz, Anna Palau, Marcus Buschbeck, Katharina S. Götze

**Affiliations:** Josep Carreras Leukaemia Research Institute (IJC), Campus ICO—Germans Trias i Pujol, Campus Can Ruti, Badalona, Spain; Department of Medicine III, Klinikum rechts der Isar, Technische Universität München, Ismaninger Strasse 22, Munich, Germany; German Cancer Consortium (DKTK) and German Cancer Research Center (DKFZ), Heidelberg, Germany

**Keywords:** Azanucleoside, Azacitidine, Decitabine, MDS, AML, HMA, Methylation, Chromatin

## Abstract

The azanucleosides azacitidine and decitabine are currently used for the treatment of acute myeloid leukemia (AML) and myelodysplastic syndromes (MDS) in patients not only eligible for intensive chemotherapy but are also being explored in other hematologic and solid cancers. Based on their capacity to interfere with the DNA methylation machinery, these drugs are also referred to as hypomethylating agents (HMAs). As DNA methylation contributes to epigenetic regulation, azanucleosides are further considered to be among the first true “epigenetic drugs” that have reached clinical application. However, intriguing new evidence suggests that DNA hypomethylation is not the only mechanism of action for these drugs. This review summarizes the experience from more than 10 years of clinical practice with azanucleosides and discusses their molecular actions, including several not related to DNA methylation. A particular focus is placed on possible causes of primary and acquired resistances to azanucleoside treatment. We highlight current limitations for the success and durability of azanucleoside-based therapy and illustrate that a better understanding of the molecular determinants of drug response holds great potential to overcome resistance.

## Background

### Historical view of azanucleosides

Azanucleosides (AZN) are pyrimidine analogues of the nucleoside cytidine that were originally developed as classical cytostatic agents to be used at higher doses [1]. Nowadays, these compounds are known to be potent inhibitors of DNA methylation when used at lower doses and are often referred to as hypomethylating agents (HMAs) [2]. As such, AZN are considered the first epigenetic drugs. Azanucleosides currently used in clinic are 5-azacytidine (azacitidine) and 5-aza-2′-deoxycytidine (decitabine).

AZN were first synthesized in 1964 [1] and clinical trials examining their anticancer activity commenced as early as 1967 [3]. In 1978, it was shown that AZN are able to induce terminal differentiation of mouse embryo cells, which was later associated with changes in DNA methylation [2, 4]. This was the first link of DNA methylation to cellular differentiation. As patterns of methylation and chromatin structure are known to be severely altered in many forms of cancer, often with gene silencing of key tumor suppressor genes as a result of promoter hypermethylation, this important observation opened an avenue for developing DNA methylation inhibitors for cancer treatment. In particular, acute leukemias as well as myelodysplastic syndromes had been shown to exhibit multiple silenced tumor suppressor genes, one reason why AZN were tested in these diseases. Importantly, in the initial experiments of Jones and Taylor [2, 4], optimal inhibition of DNA methylation and subsequent differentiation of cells was observed at lower drug concentrations with prolonged exposure, whereas higher concentrations led to a decrease in demethylation and differentiation. The original studies of decitabine and azacitidine in the 1980s for the treatment of leukemia employed these drugs at the maximum tolerated dose (e.g., 2500 mg/m^2^ decitabine per course) but had to be discontinued due to prolonged myelosuppression. Subsequently, both drugs were explored at much lower doses allowing optimal hypomethylation as suggested by Jones and Taylor. This led to the design of the first successful clinical trial in myelodysplastic syndromes using low doses of azacitidine (75 mg/m^2^) administered over a prolonged period of 7 days repeated every 28 days, demonstrating superiority over best supportive care [5]. On the basis of these data, azacitidine was approved by the US Food and Drug Administration (FDA) in 2004 for the treatment of myelodysplastic syndromes (MDS) and thus is the first HMA agent approved for treatment of this disease [6]. Similarly, decitabine was shown to have activity in higher-risk MDS at a low-dose schedule of 15 mg/m^2^ every 8 h for 3 days repeated every 6 weeks [7]. Decitabine was approved by the FDA for the treatment of MDS using this schedule in 2006. A lower-dose regimen with higher-dose intensity of 20 mg/m^2^ over 5 days repeated every 28 days was later suggested as a superior regimen within a randomized phase III trial [8].

## Molecular drug action

**Fig. 1:**
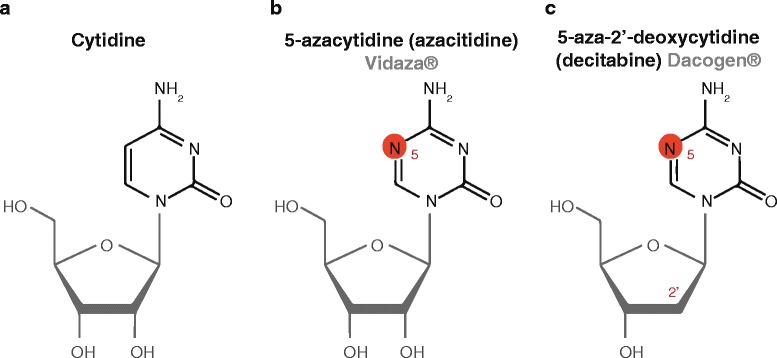
Cytidine nucleoside (**a**) and azanucleoside (**b**, **c**) chemical structures. Sugar moieties are indicated in *grey* and chemical changes between cytidine nucleoside and azanucleosides are highlighted in *red*

AZN are analogues of the naturally occurring pyrimidine nucleoside cytidine (Fig. 1) and have so far been shown to have two main mechanisms of antitumor activity: (i) cytotoxicity due to incorporation into DNA (and RNA for azacitidine) leading to induction of DNA damage response and (ii) DNA hypomethylation through inhibition of DNA methyltransferase, enabling restoration of normal growth and differentiation. Although azacitidine and decitabine are considered to be mechanistically similar drugs, they have also been shown to exhibit distinctly different effects and have shown varied clinical efficacy in clinical trials. Some of the differences in efficacy may stem from dosing issues as well as differences in incorporation into RNA and DNA as delineated below, while others may have to do with the specific disease characteristics of the treated patients within each trial.

**Fig. 2:**
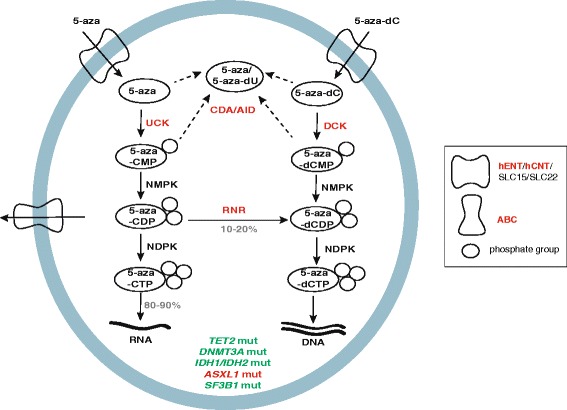
Azanucleoside uptake and intracellular metabolism. Human equilibrative and concentrative nucleoside transporters (hENT/SLC29A and hCNT/SLC28A, respectively) and the SLC15 and SLC22 transporter families mediate azanucleosides (5-aza and 5-aza-dC) uptake. Once inside the cell, the drugs are activated through consecutive ATP-dependent phosphorylation steps: the first one is mediated by uridine-cytidine kinase (UCK) for 5-aza and by deoxycytidine kinase (DCK) in the case of 5-aza-dC; the enzyme nucleoside monophosphate kinase (NMPK) incorporates the second phosphate group in both drugs; then, ribonucleotide reductase (RNR) partly converts (10–20 %) 5-aza-CDP into its deoxy form 5-aza-dCDP. Finally, nucleoside diphosphate kinase (NDPK) adds the third phosphate group and 5-aza-CTP is incorporated into RNA while 5-aza-dCTP is incorporated into DNA. Enzymes involved in resistance are highlighted in *red*, while mutated genes, which have been described to increase the sensitivity to AZN treatment or improve overall survival in patients, are highlighted in *green*

After cellular uptake of AZN by human equilibrative and concentrative nucleoside transporters 1 and 2 [9], azacitidine and decitabine are modified by different metabolic pathways to achieve their active forms (Fig. 2). The first limiting step in this cascade is the ATP-dependent phosphorylation of the nucleoside to the monophosphorylated nucleotide catalyzed by uridine-cytidine kinase for azacitidine and deoxycytidine kinase for decitabine [10]. Subsequent phosphorylation by two different kinases yields the active metabolites 5-aza-CTP for azacitidine or 5-aza-dCTP for decitabine. During replication, decitabine-derived 5-aza-dCTP is incorporated in newly synthesized DNA. In contrast, 80–90 % of azacitidine is incorporated in RNA as 5-aza-CTP, while 10–20 % are incorporated into DNA after multistep conversion to 5-aza-dCTP by the enzyme ribonucleotide reductase (Fig. 2).

### DNA demethylation

At lower doses, DNA incorporation of 5-aza-dCTP impairs DNA methylation by irreversible inhibition of DNA methyltransferases (DNMT), particularly DNMT1, which is responsible for the maintenance of methylation following DNA replication [11]. Effects on other DNMTs, such as DNMT3A and DNMT3B, have only been observed at higher AZN doses [12]. DNMT1 recognizes incorporated 5-aza-dCTP as natural substrate and becomes irreversibly bound to the cytidine analogue, inhibiting DNA methyltransferase function and leading to its degradation [13]. As a consequence, methylation marks become lost during DNA replication. This hypomethylation of DNA can then lead to re-activation of silenced tumor suppressor genes [14].

Covalent DNMT-5-aza-dCTP-DNA adducts have also been shown to induce DNA damage ATM/ATR response pathways resulting in growth inhibition, G2 cell cycle arrest, and apoptosis [15]. This activation is represented by strong induction of γ-H2AX and activation of DNA repair proteins including CHK1, CHK2, and RAD51 [16]. The DNA damage caused by AZN is repaired by the base excision repair (BER) machinery and is susceptible to inhibition of poly-ADP ribose polymerase (PARP) [17]. As DNMT1 has also been demonstrated to have a role in DNA repair, inhibition of DNMT1 by AZN may also indirectly influence DNA repair mechanisms [18]. Recent findings support this by showing that depletion of DNMT1 by RNA interference in combination with AZN treatment can reduce AZN-induced DNA damage formation, thereby decoupling DNA damage and DNA demethylation in response to AZN [19]. This further highlights that other mechanisms, besides DNMT inhibition, are involved in AZN action.

### RNA-dependent effects

Since most of the drug azacitidine is incorporated into RNA, it is assumed that its efficacy, at least in part, is due to RNA-dependent effects, which are cell-cycle independent. Incorporation of 5-aza-CTP into RNA inhibits tRNA methylation and processing [20] by reducing tRNA methyltransferase levels [21], giving rise to defective messenger and transfer RNAs. Furthermore, it disrupts rRNA processing ultimately leading to inhibition of mRNA and protein synthesis and thus inducing apoptosis [22]. Recently, it has been shown that RNA incorporation of 5-aza-CTP is able to repress the expression of the M2 subunit of ribonucleotide reductase (RRM2) and therefore interferes with the conversion of ribonucleotides to deoxyribonucleotides, leading to inhibition of DNA synthesis and repair [23]. As decitabine is not incorporated into RNA, it does not have any direct effects on RNA processing.

### Immune effects

In addition to their demethylating activity, AZN have recently been shown to induce specific immune responses in cancer cells [24, 25, 26]. Analysis of expression changes in response to azacitidine treatment in different cancer cell lines (breast, colorectal, and ovarian) led to the identification of a subset of azacitidine-induced immune genes (AIM), which can be used to classify primary tumors into low and high expression groups. For the latter, treatment with azacitidine combined with immunotherapy has been suggested to be beneficial [25]. In addition, it has been demonstrated that treatment with DNA methylation inhibitors sensitizes murine melanoma cells to anti-CTLA4 immunotherapy [24], suggesting that a treatment combination of AZN and PD1/PDL-1 antibodies may show synergistic effects [25].

A recent study on ovarian cancer has provided an intriguing molecular mechanism linking AZN treatment to the immune response. DNA methylation inhibitors were shown to upregulate endogenous retroviruses in tumor cells, leading to a tumor-inhibiting immune response. This type I interferon immune response is thought to be a result of induction of the viral defense pathway, which detects cytosolic double-stranded RNA originating from endogenous retrovirus genes (ERVs) re-expressed upon AZN treatment [24]. However, if a similar mechanism also occurs in hematologic cancers in response to AZN treatment will need to be determined.

In addition to inhibition of DNA methylation and RNA metabolism, AZN also inhibits the nuclear factor kappa B (NFkB) pathway through inhibition of phosphorylation of the NFkB-activating kinase IKKalpha/beta [27]. As a consequence, AZN can specifically inhibit regulatory T cells in MDS patients [28].

Furthermore, azacitidine has been reported to impair de novo synthesis of pyrimidine through inhibition of uridine monophosphate synthase, leading to a significant decrease in UTP and CTP levels [29], which results in impairment of cholesterol and lipid homeostasis and contributes to the cytostatic effect of azacitidine [30].

### Differences between azacitidine and decitabine

AZN are most toxic during S-phase of the cell cycle [31], and while cytotoxicity requires relatively high doses, the effect on DNA methylation preferentially occurs at lower doses [32]. AZN are intrinsically instable and after administration undergo spontaneous hydrolysis as well as deamination by cytidine deaminase leading to degradation [33]. At low doses, the effects of AZN are rapidly lost upon drug withdrawal and therefore continuous administration and several treatment cycles are required to sustain response [34]. The maximum plasma concentration of active AZN-derived metabolites achieved by standard treatment is 3–11 μM for azacitidine or 0.5 μM for decitabine [35, 36, 37]. These concentrations have been shown to induce transient demethylation in various hypermethylated loci in patients and thus provide valuable proof of mechanism [37, 38]. However, a clear correlation between the extent of genome-wide DNA hypomethylation, re-expression of tumor suppressor genes, and clinical response to AZN has not been demonstrated thus far.

Due to its primary incorporation into newly synthesized DNA, decitabine has been shown to be more potent than azacitidine in vitro, leading to inhibition of DNMT1, DNA hypomethylation, and DNA damage induction at concentrations two- to tenfold lower than azacitidine [10, 39, 40, 41]. Unlike azacitidine, decitabine is an S-phase specific agent that only targets proliferating cells in S-phase while other stages of the cell cycle remain unaffected [42]. Taking into account the short half-life, to improve treatment success, shorter intervals of decitabine treatment or continuous infusion are needed to increase the probability that all cancer cells enter S-phase and thus the therapeutic time window [43]. Hypomethylation after decitabine treatment is dose dependent, peaking at 10–15 days after administration and recovering to baseline at 4–6 weeks [44]. Although preclinical data suggested that continuous infusion of decitabine may be advantageous, dose-finding trials indicated that short bolus infusions in patients with a poor hematological status may be better than continuous infusion, lower doses are better than higher doses, and dose intensity is important for efficient DNA demethylation [8, 45]. These observations led to the current standard dosing schedule of 20 mg/m^2^ per day over 5 days. However, because this trial had only a small number of patients in the dosing regimens deemed as inferior, novel continuous dosing schedules merit clinical investigation. In contrast to decitabine, azacitidine is administered subcutaneously at a dose of 75 mg/m^2^/day over 7 days. Other schedules of azacitidine (e.g., over 5 or 10 days) have also been examined but not proven superior.

Importantly, studies examining gene expression in human acute myeloid leukemia (AML) cell lines showed that patterns of drug-modulated gene expression were non-overlapping between azacitidine and decitabine [46], indicating that both drugs indeed may have different target genes. This observation in addition to the biochemical differences may explain why one drug may work in a patient while the other may not.

## Current status of clinical application of hypomethylating agents in hematologic cancers

Azacitidine (Vidaza^®^) and decitabine (Dacogen^®^) were first tested for the treatment of MDS decades ago with the first large randomized trial published in 2002 [5] and have emerged as very promising drugs in this field. MDS are a heterogeneous group of clonal hematopoietic stem cell disorders characterized by dysplastic and ineffective hematopoiesis with clinically apparent cytopenias in the peripheral blood and a predisposition to AML. Primarily a disease of the elderly, treatment options are limited due to comorbidities and a high progression rate into secondary AML in one third of the patients [47]. The only proven curative approach for MDS is allogenic stem cell transplantation (allo-SCT), which is not a feasible option in the majority of MDS patients.

Since the FDA approval of azacitidine for all MDS subtypes in 2004 and the positive results of several clinical trials, the drug has been widely used in the clinic [5, 47, 48] and has become the standard of care for higher-risk MDS patients (IPSS intermediate-2 and high or IPSS-R very high and intermediate) not eligible for allo-SCT [48, 49]. The international randomized phase III multicenter “AZA-001” trial revealed that patients benefit from hematologic improvement (47 %) and a significant delay in progression to AML as well as a prolonged survival under azacitidine treatment vs. conventional care regimens with a median overall survival 24.5 vs. 15 months [48]. These results make azacitidine the first drug to confer a survival benefit to MDS patients. Most responders achieve a first response within 6 cycles of subcutaneous azacitidine injection (75 mg/m^2^ per day for 7 days every 28 days), and continuation of treatment after first response improves response quality [50]. Treatment continuation is recommended until progression of the disease or unacceptable toxicity [51].

Ongoing studies are currently examining the efficacy of oral azacitidine which shows a higher bioavailability [52, 53]. The oral formulation (maximal tolerated dose 480 mg) is under investigation to serve as a long-term low-intensity treatment option (extended to 14 or 21 days cycles) for lower-risk MDS with additional poor prognostic factors such as severe thrombocytopenia (AZA-MDS-003 trial) or as a post-transplant maintenance therapy for MDS as well as AML patients (CC-486-AML-002 trial) [53, 54, 55].

In contrast to the results of the AZA-001 study for azacitidine, clinical trials for decitabine have thus far not been able to show an overall survival benefit in patients with MDS. Initial phase II trials in Europe using decitabine at a total dose of 225 mg/m^2^ as a continuous infusion over 72 h or at a total dose of 135 mg/m^2^ using a 3-day intravenous administration over 4 h three times a day showed a favorable overall response rate between 49 and 54 %, comparable to that of azacitidine. In a randomized phase III trial testing decitabine in a schedule of 15 mg/m^2^ for a total of nine doses over 72 h compared to best supportive care, the overall response rate was 30 % [7]. Patients receiving decitabine had a prolonged median time to progression to AML or death compared to patients receiving best supportive care, but overall survival was not improved. The drug has therefore not been licensed for treatment of MDS in Europe so far, although it is approved in the USA in this indication. Results of a schedule which can be administered in the outpatient setting using 20 mg/m^2^ decitabine as intravenous infusion for five consecutive days yielded comparable clinical efficacy [56].

Based on the encouraging results of the MDS trials for hypomethylating agents, these drugs are also being evaluated for the treatment of AML, in particular or patients not eligible for intensive chemotherapy. As incidence of AML increases with age and our population grows increasingly older, the median age at diagnosis is now approximately 70 years. Treating elderly AML patients presents a significant therapeutic challenge as intensive treatment is often not possible due to comorbidities and poor performance status. In addition, AML of the elderly more often exhibit adverse cytogenetic and molecular genetic features leading to poor outcome after intensive chemotherapy. Thus, there is an urgent need for effective but less toxic therapies for elderly AML patients.

First data on the efficacy of azacitidine in AML emerged from the AZA-001 trial as a significant percentage of patients in the AZA-001-MDS trial were originally classified as the advanced MDS subtype RAEB-t with 20–30 % bone marrow blasts. This subgroup no longer exists in the WHO classification of MDS and is now considered as AML which is defined by 20 % or more blasts in the bone marrow. A separate analysis of this AML subgroup within the AZA-001 trial showed that these patients benefitted from a significantly improved overall survival compared to conventional care [57]. Similarly, azacitidine treatment has been associated with a median overall survival of approximately 9 to 10 months in patients with AML who participated in the Austrian Azacitidine Registry or in a French compassionate use program [58, 59]. These encouraging results paved the way for a large randomized phase III trial in AML patients older than 65 years and with bone marrow blast counts >30 % (AZA-AML-001). Patients with AML were randomized to receive either azacitidine (75 mg/m^2^ days 1–7 q28) or investigator’s choice of conventional care (CCR, consisting of BSC, low-dose ARA-C or intensive chemotherapy) [60]. Although this trial did not reach its primary endpoint, the results showed an improved overall survival for patients treated with azacitidine compared to conventional care (10.4 vs. 6.5 months, *p* = 0.1). Remission rates were not increased in the azacitidine arm compared to CCR but the subgroup of patients with adverse cytogenetics or AML with myelodysplasia-related changes showed a significantly improved overall survival. This trial demonstrates that azacitidine is superior to CCR in biologically poor-risk subgroups and makes azacitidine the only drug so far to show a survival benefit in elderly AML patients with adverse cytogenetics [60]. Based on the positive results of the AZA-AML-001 trial, azacitidine was licensed for the treatment of AML in patients older than 65 years in 2015. Azacitidine was also evaluated as an addition to standard intensive chemotherapy with the cytotoxic agents daunorubicin and cytosine arabinoside (ARA-C) for the treatment of elderly AML patients older than 60 years but showed increased toxicity and failed to improve overall survival for unselected patients [61], although patients with mutations in DNMT3A seemed to derive a survival benefit from the addition of azacitidine (Müller-Tidow, personal communication).

Similarly, decitabine was also evaluated for the treatment of elderly AML in a large phase III randomized trial (DACO-16), where the drug was tested against BSC or low-dose ARA-C [62]. Decitabine significantly improved response rates compared to BSC/low-dose ARA-C (17.8 vs. 7.8 months, *p* = 0.001) but overall survival was not significantly increased 7.7 vs. 5 months, *p* = 0.1). Nevertheless, decitabine was licensed for the treatment of elderly AML in Europe in 2013. It is currently not approved in this indication in the USA.

A remarkable clinical hallmark of both hypomethylating agents is that they achieve significant clinical efficacy and can confer a survival benefit in MDS and AML despite inducing only low complete remission rates of around 20 %. This challenges the long-standing dogma that achievement of complete remission is necessary for improvement of overall survival. It also further demonstrates that we lack a sufficient understanding of the systemic and cellular actions of these drugs, which might involve still unrecognized molecular mechanisms.

### Differences in outcome between azacitidine and decitabine trials

A direct comparison between azacitidine and decitabine in terms of efficacy within a controlled clinical trial has not been performed so far. It is therefore difficult to accurately assess why the survival outcomes in the randomized MDS trials were so different between both drugs. Although remission rates were similar for azacitidine and decitabine, the overall survival in the experimental arm was significantly shorter in the decitabine trial compared to the azacitidine trial [7, 48]. Besides the possibility that this may be due to differing mechanisms of action, it is also clear that the patient characteristics in the two large randomized MDS trials were divergent. In addition, patients enrolled in the decitabine trial received a maximum of 8 cycles of therapy (median 4 cycles) while patients in the azacitidine trial were treated until progression (median 9 cycles). A detailed comparison of patients’ characteristics in both trials is shown in Table 1 and the current approval status for azacitidine and decitabine in MDS and AML in Table 2. The more direct metabolic activation pathway to DNA incorporation of decitabine compared to the primarily RNA incorporation of azacitidine has been suggested to make decitabine a more potent compound than azacitidine. A large retrospective matched-pair analysis of patient outcomes in 300 patients treated with either azacitidine or decitabine suggested that while remission rates were similar between the two drugs, overall survival was decreased in patients >65 years treated with decitabine compared to azacitidine [63]. This seemed primarily due to an increased infection rate in patients receiving decitabine, perhaps due to higher cytotoxicity and possibly an additional contributing factor to the inferior outcome in terms of overall survival.

**Table 1:** Comparison of patient characteristics between the AZA-001 trial and the EORTC 0611 trial for high-risk MDS

	AZA-001 trial: azacitidine	EORTC 0611 trial: decitabine
Eligibility criteria	IPSS INT2/high	IPSS INT1/INT2/high
	MDS with 5–30 % blasts	MDS with 11–30 % blasts or <10 % blasts and poor cytogenetics
	CMML with >10 % blasts and WBC <13 G/L	CMML independent of blast counts or WBC counts
	No t-MDS allowed	t-MDS allowed
Treatment schedule	75 mg/m^2^ days 1–7, q28	15 mg/m^2^ 3× day q42
	Treatment until progression	Maximum number of 8 cycles
Patient cohort		
IPSS high	46 %	38.70 %
Poor cytogenetics	28 %	48 %
t-MDS	0	12.60 %
Median cycle number	9	4

**Table 2:** Approval status for azacitidine and decitabine in MDS and AML

	Azacitidine (Vidaza)		Decitabine (Dacogen)	
	MDS	AML	MDS	AML
USA (FDA)	All subtypes	AML 20–30 % blasts (formerly RAEB-t)	All subtypes	AML <30 % blasts (formerly RAEB-t)
Dose	75 mg/m^2^ s.c. days 1–7 q28	75 mg/m^2^ s.c. days 1–7 q28	15 mg/m^2^ i.v. 3× daily days 1–3 q42 or 20 mg/m^2^ i.v. days 1–5 q28	15 mg/m^2^ i.v. 3× daily days 1–3 q42 or 20 mg/m^2^ i.v. days 1–5 q28
Europe (EMA)	INT2/high-risk MDS according to IPSS, CMML 10–29 % blasts, not eligible for allogeneic SCT	AML ≥65 years regardless of blast counts, not eligible for allogeneic SCT	Not approved	AML ≥65 years not candidates for standard induction chemotherapy
Dose	75 mg/m^2^ s.c. days 1–7 q28	75 mg/m^2^ s.c. days 1–7 q28	n/a	20 mg/m^2^ i.v. days 1–5 q28

## Limitations of the treatment and possible causes of acquired and primary resistance

The clinical outcome of patients with MDS is quite variable even within the same WHO subtype and when classified according the revised IPSS score, which has become the standard prognostic model in MDS [47]. Since every second patient has no clinical benefit from azacitidine treatment and at least 4 to 6 cycles are required before treatment failure becomes apparent [50], the development of response predicting biomarkers is an unmet need for improving patient selection and designing better therapeutic options.

In the past few years, a large number of recurring somatic gene mutations have been discovered in patients with MDS [64, 65, 66]. First studies analyzing the mutation status of a panel of genes failed to provide predictive markers that would allow identification of responders prior to treatment initiation with hypomethylating agents [67]. Moreover, higher-risk MDS patients frequently progress to AML even when therapy is continued [48]. Eventually, all patients including those initially achieving remissions or hematologic improvement develop resistances to AZN leading to treatment failure [48]. These failures can be divided into two broad categories: refractory or primary AZN failure, which is seen in patients that show no response to at least 6 cycles of therapy, and secondary failure in patients that either progress under treatment or relapse after termination of treatment. Although the processes responsible for both primary and secondary AZN failures have been a topic of intense research, the molecular mechanisms of resistance are not well understood.

First causes of AZN resistance have been found both in the metabolic pathways that activate AZN and pathways linked to DNA methylation. A recent study analyzing decitabine response in MDS patients described alterations in nucleoside metabolism leading to difference in response [68]. In this study, 32 responders and non-responders as well as 14 patients showing complete remission and subsequent relapse were examined. In responders, the ratio of cytidine deaminase (CDA) to deoxycytidine kinase (DCK) was higher compared to non-responders [68]. This suggests that increased deamination and decreased phosphorylation of decitabine is able to confer primary resistance to the drug, although the exact mechanisms leading to a changed CDA/DCK ratio are not known. Similar results were obtained in patients treated with azacitidine, in which the expression of uridine cytidine kinase was lower in patients without a response than in patients responding to azacitidine treatment [69].

Downstream of the AZN action, several gene mutations have been proposed to affect treatment outcome. For example, several groups have found that mutations in *TET2*, an enzyme that converts 5-methylcytosine to 5-hydroxymethylcytosine correlated with increased sensitivity to AZN treatment [70, 71, 72]. This correlation was more significant in the absence of *ASXL1* mutations [70]. Despite correlation with response, mutated *TET2* was not associated with improved overall survival [70, 72]. Furthermore, these studies could not identify a mutational pattern associated with the absence of response and thus the presence of specific mutations cannot be used to identify non-responders. Mutations in other genes involved in epigenetic regulation, such as *DNMT3A*, *ASXL1*, and *IDH1*/*IDH2*, have also been suggested to affect AZN response [72]. Moreover, the expression of *BCL2L10*, a member of the Bcl2 family preventing cell apoptosis, positively correlates with AZN resistance [73].

Despite these reports, efforts to correlate response to AZN with hypermethylation patterns have not yielded clear-cut results. None of the studies were able to reveal clinical or molecular patterns to identify non-responders, and no diagnostic tool can yet be used as a basis for forgoing HMA treatment. Neither the measurement of pre-treatment DNA methylation [66] nor the presence of *TP53* mutations is predictive for response to azacitidine in MDS, although as expected *TP53* mutated patients have a poor overall survival despite response [74]. *ASXL1*-mutated clones seem to mediate a partial resistance to azacitidine, as they showed a tendency to lower likelihood of response [70]. But even in responding patients, the drug is not curative and response is lost over time. Recently, Meldi et al. identified 167 differentially methylated DNA regions at baseline in patients with the subtype of chronic myelomonocytic leukemia (CMML) that could predict response to decitabine at the time of diagnosis [75]. This study also showed that upregulation of the cytokines CXCL4 and CXCL7 may contribute to primary decitabine resistance, as these molecules were overexpressed in non-responding patients. Ongoing studies are currently investigating similar correlations between methylation status and response in MDS.

It has been shown that azacitidine does not eradicate the leukemia stem cell (LSC)-containing population even in patients achieving a complete remission. A later expansion of this LSC-containing population leads to relapse [76] and thus provides a possible explanation for AZN treatment failure over time. Recently, it was also shown for CMML that the mutation allele burden in responding patients remains stable during treatment with AZN despite changes in DNA methylation and gene expression [77]. These data indicate that hematologic response is primarily due to epigenetic modulation rather than eradication or suppression of the leukemic clone. Why this epigenetic modulation fails to be effective over time is not understood at present and is the focus of ongoing research efforts. Acquired resistance to AZN may conceivably also be due to acquisition of new gene mutations over time or a growth advantage of primary resistant clones such as those with mutant *ASXL1*. These hypotheses are currently being examined in several studies analyzing serial bone marrow samples of patients over time as well as other efforts developing and analyzing novel murine models of MDS.

## Unmet needs and ongoing clinical trials

MDS patients failing treatment with AZN have a very poor outcome with a median survival of 5.6 months [78]. Switching the AZN agent at progression (i.e., from azacitidine to decitabine or vice versa) is an option that has been described as successful in some cases although extensive data is lacking [79]. Unmet medical needs are therefore improvement of response rates and response duration for patients receiving AZN as well as development of alternative therapies after failure of AZN.

To this end, the effectiveness of several combination therapies is currently being investigated. Since DNA methylation and hypoacetylation often occur at the same time and ensure robust inhibition of gene expression, the combination of AZN with histone deacetylase (HDAC) inhibitors has been proposed to improve treatment outcome. Indeed, in vitro experiments combining AZN with HDAC inhibitors have shown synergistic re-expression of a subset of genes [80]. Based on this observation, several clinical trials have evaluated the combination of AZN with HDAC inhibitors, and while some had promising results, others demonstrated significant toxicity (reviewed in [81]). Thus, combination therapy with HDAC inhibitors requires further investigation. Currently, combination therapies of mocetinostat (NCT02018926) or pracinostat (MEI-005, NCT01993641) with azacitidine are being tested.

Azacitidine and decitabine have also been tested in combination with (i) immunomodulatory drugs (lenalidomide, monoclonal antibodies) or (ii) chemotherapy. Simultaneous administration of azacitidine and lenalidomide was highly effective in higher-risk MDS patients with an overall response rate of 72 % [82]. The best response was observed in patients with at least one mutation in *TET2*, *IDH1/2*, or *DNMT3A* but also induced significantly higher toxicity [82]. Decitabine in combination with the monoclonal antibody gemtuzumab has shown improved response rates in MDS and AML patients compared to historical controls [83]. Furthermore, next-generation epigenetic agents, such as other DNMT inhibitors, compounds directly targeting mutated or dysregulated proteins, including Idh1, Idh2, Ezh2, and Brd2/4, as well as kinase inhibitors (rigosertib, volasertib) [84] and immune checkpoint inhibitors (PD-1/PD-L1) are currently being tested [85].

Ongoing phase II/III trials are also examining the administration of azacitidine for the prevention or treatment of relapse in patients after hematopoietic stem cell transplantation (RELAZA trial, NCT01462578). Also, the oral formulation of azacitidine is currently being tested in a phase III trial for continuous administration and extended low dose schedules as a maintenance therapy in AML (Quazar AML-001 trial, NCT01757535) as well as for lower-risk MDS patients with low platelet counts (AZA-MDS-003 trial, NCT01566695).

In addition to myeloid malignancies, azacitidine is also being investigated in lymphoid malignancies such as relapsed aggressive B-cell lymphomas (DLBCL-001 trial, NCT02343536) or T-cell lymphomas in combination with chemotherapy and other agents such as proteosome inhibitors like bortezomib or HDAC inhibitors like romidepsin.

## Hypomethylating agents for treatment of solid tumors

Due to its promising results in hematologic malignancies, AZN are further being tested in phase I/II clinical trials for advanced solid tumors—mainly colorectal cancer, small-cell lung carcinomas, ovarian cancer, and breast cancer. Low-dose decitabine in combination with cytotoxic drugs has shown encouraging results with a response rate up to 60 % [86]. Furthermore, combination of low-dose azacytidine with the HDAC inhibitor entinostat in refractory advanced non-small cell lung cancer led to impressive responses in a subset of patients [87].

A detailed description of epigenetic therapy (including AZN) in solid tumors has recently been reviewed [88].

## Conclusions

AZN have provided a significant improvement in the treatment of higher-risk MDS and elderly AML. However, while they show significant efficacy, these patients continue to have an overall poor prognosis. Thus, it will be important to obtain a better understanding of the AZN action and to identify and validate biomarkers that predict treatment response as well as understand the mechanisms leading to AZN failure.

Although preclinical studies indicate that decitabine is a more potent antileukemic agent than azacitidine [40, 41], the clinical data suggest that azacitidine is more effective than decitabine. In order to elucidate this apparent contradiction, future investigations of decitabine should be performed to optimize the current dose schedule.

Certainly, it has become clear that single-agent AZN treatment is insufficient for achievement of long-term remissions, and therefore, the suitability and effectiveness of combining AZN with other drugs needs to be investigated in order to find novel strategies to improve treatment success and its durability for patients.
